# CRISPR-Cas9 mediated editing of *starch branching enzyme*, *SBE2* gene in potato for enhanced resistant starch for health benefits

**DOI:** 10.3389/fgeed.2025.1686412

**Published:** 2025-11-26

**Authors:** Sudha Batta, Sundaresha Siddappa, Neha Sharma, Rajender Singh, Reena Gupta, Dinesh Kumar, Brajesh Singh, Ajay Kumar Thakur

**Affiliations:** 1 Department of Biotechnology, Himachal Pradesh University, Shimla, Himachal Pradesh, India; 2 ICAR-Central Potato Research Institute, Shimla, Himachal Pradesh, India; 3 ICAR-Indian Institute of Wheat and Barley Research, Karnal, Haryana, India

**Keywords:** potato, high amylose, resistant starch, CRISPR-Cas9, starch branching enzymes, SBE2.1 & SBE2.2

## Abstract

Potato is an important vegetatively propagated, starch-rich tuber crop. High amylose potatoes containing more resistant starch offer healthier food alternatives. However, the resistant starch content is low in most cultivated potato varieties. In this study, targeted mutation of the *starch branching enzyme2* (*SBE2.1* & *SBE2.2* isoforms) had been done in the commercially significant potato cultivar, Kufri Chipsona-I using Clustered regularly interspaced short palindromic repeats-CRISPR-associated protein 9 (CRISPR-Cas9 system) to develop high-amylose potato lines. *SBE2* is one of the key enzymes involved in amylopectin biosynthesis, a starch component. Two isoforms, *SBE2.1 & SBE2.2,* were mutated using CRISPR-Cas9-mediated genome editing. After *Agrobacterium-mediated* genetic transformation, fifty transformed lines were generated on herbicide Basta selection medium, out of which 70% were found positive for *bar* and *Cas9* genes. Overall, six mutant lines, *viz.* K301, K302, K303, K304, K305, K306, derived from distinct events, exhibited deletions and substitutions in the target exons. The CRISPR-Cas9 edited K304 potato line exhibited both insertion–deletion (indel) and substitution mutations in three out of the four selected targets across both genes, and was therefore identified as the most efficiently edited line. The harvested tubers from *SBE2.1 & SBE2.2* mutant K304 line showed the highest amylose (95.91%) and resistant starch content (8.69 g/100 g). Evaluation of starch using X-ray crystallography (XRD) illustrated an altered crystallinity index (CI%) in all six mutant events in comparison to the wild study. Furthermore, ^1^H-NMR study demonstrated a substantial decline in branch chain elongation in amylopectin, and thus a low degree of branching in a range of 1.15%–3.66% was reported in mutant lines, relative to the wild type (5.46%). The present study demonstrated the efficacy of CRISPR-Cas9-mediated mutagenesis of starch biosynthetic genes to develop high-amylose potato lines with elevated resistant starch content for improved health benefits.

## Introduction

Potato (*Solanum tuberosum* L.) is a starchy, non-grain tuber crop that belongs to the *Solanaceae* family. Potatoes are the third-largest food crop in the world after rice and wheat. It is one of the major crops that contributes to global food and nutritional security. Potatoes are cultivated in more than 100 countries and are a major component of the world’s daily diet. India is the second-largest potato producer after China, with an average productivity of 28.24 tons per hectare in the year 2023, which is higher than world’s average of 25.13 tons per hectare (Hu et al., 2025). The domestic demand for potatoes in India is mainly for their use as raw material for home meals, food industries, and as a seed stock for multiplication and propagation (Mukherjee, 2025). Potatoes are a rich source of carbohydrates, vitamin C, potassium, and dietary fibres. Thus, popularly known as ‘Poor man’s food’, however, the major concern associated with potato consumption is its high glycemic index and its impact on human health (Timar et al., 2024).

Potato tubers contain ⁓26% starch, of which 70%–80% amylopectin (highly digestible) and 20%–30% amylose (slow digestible), with 3% of proteins, 1% of vitamins, and the remaining 70% is water (Rosell and Nystrom, 2024). The α-1,6-glucosidic linkages provide branching points in both amylose and amylopectin biopolymers and are scattered throughout the clusters of alternating linear α-1,4-glucan chains, which is the key feature that differentiates amylopectin from amylose (Apriyanto et al., 2022). Amylose on heating re-associates rapidly to form a precipitate and then turn into a gel upon cooling, which is resistant to digestion by α-amylases. Whereas, amylopectin reassociates slowly, breaks down in the upper stomach, and spikes the blood glucose. Thus, high-amylose starch is a form of resistant starch that acts as dietary fiber in potato tubers. Resistant starch reduces hyperglycaemia by slowly releasing glucose into the bloodstream (Biswas et al., 2023). The resistant starch lowers the risk of diet-related disorders and promises the great health attributes by developing resistant starch-rich potato cultivars for consumers. There are two different pathways for biosynthesis of amylose and amylopectin. *Granule-bound starch synthase* (*GBSS*) is the main enzyme required for amylose biosynthesis, whereas different isoforms of *starch synthase* (*SS*), and *SBEs* are involved in amylopectin synthesis (Jayarathna et al., 2024; Sood et al., 2025). *SBEs* are the major enzymes that catalyse the transfer of α-1, 4-linked glucan chain and produce branches in amylopectin (Vishal et al., 2025).

To improve the resistant starch content in potato tubers, current research has focused on boosting the apparent amylose content of starch by targeting the genes crucial for amylopectin biosynthesis. In potato, the amount of amylopectin is determined by the activity of *SBEs,* i.e., *SBE1*, *SBE2*, and *SBE3*. Previously, several studies have altered the expression of *SBE* genes for starch modification using transgenic approaches. Jobling et al. (1999) used antisense approach to suppress the expression of *SBE1* and *SBE2* genes in potato, which led to high-amylose starch with altered structure. Similarly, Schwall et al. (2000) have also developed high-amylose potato lines with very high phosphorus content by suppressing the expression of *SBEA* and *SBEB* genes using antisense RNA technology. Further, Andersson et al. (2006) have downregulated the expression of *SBE1* and *SBE2* genes in potato using RNAi technology and developed high-amylose lines with significantly altered starch architecture. Further, Brummell et al. (2015) have demonstrated the pivotal role of *SBEII* in starch branching for improving starch quality by its overexpression through the introduction of hybrid *SBEII* carrying cDNA plus a single intron (intragene) in potato tubers. This showed that despite being a minor isoform of *SBEs*, *SBE2* exerts a major influence on starch structure and contributes to an apparent increase in amylose content.

In recent years, CRISPR-Cas9–based genome editing has emerged as a highly precise and efficient tool for introducing targeted mutations in specific genes, enabling researchers to manipulate genetic sequences with high accuracy (Sharma et al., 2025). In potato, Tuncel et al. (2019) have edited *SBE1* and *SBE2* genes for the first time using CRISPR-Cas9-mediated mutagenesis. They observed enormous increase in starch granule initiation in the mutants with strong reductions in *SBE2* gene only. In another study, Takeuchi et al. (2021) used CRISPR-Cas9 technique to knock out *SBE3* in tetraploid potato and successfully developed quadruple mutants with altered starch properties but normal growth. Zhao et al. (2021) applied DNA-free CRISPR-Cas9 to disrupt *SBE1* and *SBE2* genes in potato, resulting in starch with no branching. A similar study in rice, showed that targeted edits in *OsSBEIIb* shift amylose/amylopectin balance, elevating resistant starch and altering grain quality; multiplex edits across the starch network can amplify these effects (Sang et al., 2025). These findings highlight the predominant role of *SBE2* in starch branching in potato tubers, where it influences the amylose and resistant starch levels. However, editing of *SBE2* gene for enhancing amylose content using CRISPR-Cas9-mediated mutagenesis has not been attempted in any Indian potato cultivar. Therefore, in the present study, we have used the CRISPR-Cas9 technique to knock out two isoforms of the *SBE2* gene (*SBE2.1* & *SBE2.2*) of potato cultivar Kufri Chipsona-I to enhance the amylose production to effectively address dietary needs associated with diabetes and obesity management in the Indian population.

## Materials and methods

### Plant material

In this study, we have used the Indian potato cultivar Kufri Chipsona-1 as plant material. The *in-vitro* culture tubes of Kufri Chipsona-I were procured from the germplasm section of ICAR-Central Potato Research Institute, Shimla (HP), India.

### Design of sgRNA and development of multiplexed genome-editing construct

**FIGURE 1:**
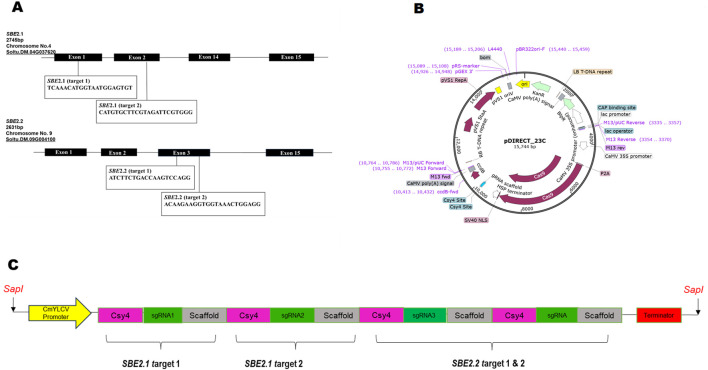
**(A)** Physical maps of the targeted regions of four sgRNAs for CRISPR-Cas9-mediated mutagenesis, **(B)** Diagram of the CRISPR-Cas9 pDIRECT_23C vector, **(C)** Schematic representation of *SBE*-sgRNA-CRISPR cassette for targeting *SBE2.1* and *SBE2.2* genes.

The sequences of *SBE2.1* (GenBank accession no. *NW_006238958.1: c2098376–2090439*) and *SBE2.2* (GenBank accession no. *NW_006238947.1: c2592132–2611729*) were retrieved from *Solanum tuberosum* cultivar DM 1–3 516 R44, Group Phureja, genome available in Potato Genome Sequencing Consortium database (http://spuddb.uga.edu/) (Xu et al., 2011). For *SBE2.1*, two target sites were selected from the conserved domains lying in exon I and exon II, while for *SBE2.2,* two gRNA targets were chosen in conserved domain region of exon III. The target sgRNAs for Cas9-mediated mutagenesis were designed using the CRISPR RGEN (http://www.rgenome.net/cas-designer) gRNA design tool (Table 1). All the selected targets were analysed using Cas-Offinder (http://www.rgenome.net/cas-offinder) and NCBI BLAST to further exclude the possibility of off-targets. The *SBE*-sgRNA-CRISPR cassette was synthetically produced with a strong promoter CmYLCV, four sgRNAs, *Csy4* endoribonuclease, scaffold region and terminator flanked by *SapI* sites on both sides and received cloned in pMK vector (Gene Art Pvt. Ltd., United States) (Figures 1A–C). This *SBE*-sgRNA-CRISPR cassette was subsequently cloned in empty pDIRECT_23C vector (Addgene Plasmid#91140) and the construct was designated as *SBE*-pDIRECT23C. This vector contained plant-optimized *Streptococcus pyogenes Cas9* protein-coding gene under the control of the cauliflower mosaic virus (CaMV) 35S promoter along with the kanamycin and Basta resistance genes for bacterial and plant selection, respectively. Further, this binary plasmid vector was introduced into the *A. tumefaciens* GV3101 strain, using the freeze-thaw method for plant transformation (Krenek et al., 2015).

**TABLE 1:** Target sgRNAs for *SBE2.1* and *SBE2.2* genes.

Sr No.	Gene	Target	sgRNA (5′-3′)
1	*SBE2.1*	target 1	TCA​AAC​ATG​GTA​ATG​GAG​TGG
		target 2	CAT​GTG​CTT​CGT​AGA​TTC​GGG
2	*SBE2.2*	target 1	ATC​TTC​TGA​CCA​AGT​CCA​GG
		target 2	ACA​AGA​AGG​TGG​TAA​ACT​GG

### Optimization of basta dosage for selection of transformants

Basta (Phosphoinothricin) tolerance in potato explants was evaluated by culturing internodal explants excised from 3 to 4 weeks old shoots of Kufri Chipsona-I plants on MS basal medium supplemented with different concentration (0, 0.5 mg/L, 1.0 mg/L, 2.0 mg/L, 3.0 mg/L, 5.0 mg/L, 10 mg/L of Basta to the autoclaved medium. Observations were recorded for callus initiation after 6–8 weeks of culturing.

### *Agrobacterium-mediated* transformation

For *A. tumefaciens*-mediated plant transformation, 0.5–1.0 cm internodal cuttings of 4-week-old Kufri Chipsona-I plants were taken as explants and pre-cultured on MS basal medium for 2 days. The explants were co-cultivated with *A. tumefaciens* GV3101 strain harbouring *SBE*-pDIRECT23C construct and regenerated on selective medium containing Basta (5 mg/L) by following the methodology given by Siddappa et al. (2023).

### Hardening of putative edited lines

The regenerated shoots of 2 – 3 cm length, with two to three leaves, were excised and sub-cultured to MS Basta (5 mg/L) selective medium. The plantlets with fully developed roots were then gently removed from culture tubes and washed with tap water to remove any residue of medium. Further, they were planted in pots containing soil/sand/cocopeat in a ratio of 1:3:2 and kept in the glasshouse for further growth and tuber formation. The well-developed tubers were harvested after 3 months.

#### Molecular analysis of transformants

##### Confirmation of putative transformants by PCR using *bar* and *Cas9* genes

The genomic-DNA from fresh young leaves was isolated from Basta-resistant plantlets and wild type Kufri Chipsona-I plants using the DNeasy Plant mini kit (Qiagen, Germany) by following the manufacturer’s instructions. The concentration and quality of the DNA were checked using a NanoDrop™ 2000 UV-VIS Spectrophotometer (Thermo Scientific^R^). The isolated DNA was visualized on a 0.8% agarose gel to assess its purity. The T-DNA integration in the transformed plants was validated by PCR using *bar* and *Cas*9 gene-specific primers (Table 2) by following the program with an initial denaturation step at 94 °C for 4 min followed by 35 cycles at 94 °C for 30 s, annealing (Tm) 56.5 °C (*bar*), 56 °C *Cas9* for 30 s and 72 °C for 30 s, and final extension at 72 °C for 4 min. The amplified DNA fragments were resolved on 1.2% agarose gel and photographed by a Gel documentation system (BioRad).

**TABLE 2:** The primers used for amplification of *SBE2.1* and *SBE2.2* gene, pDIRECT23_C, *bar* gene and *Cas9* gene.

Sr No.	Genes	Nucleotide sequence (5′-3′)	Product length [bp]	Tm (˚C)
1	Starch branching enzyme *(SBE) 2.1* (target 1)	F-CCAGAACCTGTAAACCCAAGC R-GAACACCATCATCACAGGC	487	57.4 55.2
2	Starch branching enzyme *(SBE) 2.1* (target 2)	F-CAGCTTCAAGGTATGAATAA R-GGATTCAACAGGGAAGATGG	497	50.3 54.6
3	Starch branching enzyme *(SBE) 2.2* (target 1 & 2*)*	F-GCAACTGTGTTGCTGAAGA R-GGCAGATTTCTTGCGGAAAG	646	54.8 56.0
4	pDIRECT23_C *bar* gene	F-GACAAGCACGGTCAACTTCC R-AGTCCAGCTGCCAGAAACC	416	57.5 56.2
5	pDIRECT23_C *Cas9* gene	F-GGCTGATCTCAGGCTCAT R-GAAGTGTGAGATCCTGGT	560	56.3 56.6

##### T7 endonuclease (T7EI) assay

The *SBE2.1* (targets 1 and 2) and *SBE2.2* (targets 1 and 2) gene fragments were amplified with Q5 Hot Start High-Fidelity 2X Master Mix (New England Biolabs, United States), according to the manufacturer’s protocol using the respective primer pairs (Table 2). The resulting amplified fragments (487 bp, 497 bp, and 646 bp) were purified using Qiagen QIAquick gel extraction kit. The purified PCR products were denatured at 95 °C for 5 min and re-annealed at −2 °C per second temperature ramp to 85 °C, followed by a −1 °C per second ramp to 25 °C. The annealed PCR products were incubated with T7E1 enzyme (New England Bio Labs) at 37 °C for 15 min and separated on 1.2% agarose gel.

##### Sequencing of target regions

To identify the CRISPR-Cas9 induced mutation at the sgRNA target sites, the genomic DNA of all the obtained transformed lines, as well as the wild type, were amplified with *SBE2.1* (target 1 & 2) and *SBE2.2* (target 1 & 2) forward and reverse sequence-specific primers (Table 2). These amplified gene fragments were purified using Qiagen QIAquick gel extraction kit and sequenced using BigDye™ Terminator v3.1 ready reaction mix following the cycling program with an initial denaturation step at 96 °C for 1 min followed by 25 cycles at 96 °C for 10 s, 50 °C for 5 s, and 60 °C for 4 min. The amplified products were purified using a Qiagen DyeEx 2.0 spin kit and sequenced using a Genetic Analyzer 3,500 (Applied Biosystem, United States). The mutation types and editing efficiencies of the selected gRNAs were analyzed using Synthego ICE Analysis tool (https://ice.synthego.com).

##### Gene expression analysis using quantitative real-time PCR (qRT-PCR)

For gene expression analysis, qRT-PCR of *SBE2.1* and *SBE2.2* genes of edited potato lines as well as wild type, the RNA was isolated using the plant mini kit (Qiagen). RNA concentration and quality was analysed using a Nanodrop Spectrophotometer (Thermo Scientific), and cDNA was synthesized using high-capacity cDNA reverse transcription kit (Applied Biosystems™). The qRT-PCR reaction mixture comprised of 7.5 μL SYBR green PCR master mix (Applied Biosystems™), 1 μL of respective (10 μm) forward and reverse gene specific primers (Table 3), 4.5 μL water, and 1 μL cDNA, for a total reaction volume of 15 μL. The qRT-PCR was performed in triplicate, taking the housekeeping gene *elf* (*elongation factor*) as a reference gene. Specificity of the primers was confirmed by melting curve analysis. The generated Ct values of target genes were normalized to the Ct value of the reference gene. Relative expression was calculated using the 2^−ΔΔCT^ method and expressed as fold increase with respect to control.

**TABLE 3:** Primer sequences for qRT-PCR analysis of edited potato lines.

S. No.	Genes	Primer sequence	Amplicon size	Tm (°C)
1	*SBE2.1* (target 1)	F-GCCAGTCATTCCACACAACTCCA	120 bp	63.18
		R-GCTGCAAACTTTGTGGCGTCTG		63.55
2	*SBE2.1* (target 2)	F-TCAAATACCCTCGCCCTCCCA	109 bp	63.01
		R-TCATCTGCAAACTCACGATACGA		60.12
3	*SBE2.2* (target 1 & 2)	F-AGGATCAGAGAGAGGGGCATCC	159 bp	62.77
		R-AGCTTCCAAACCACCCTCATACT		61.60
4	*elF* (reference gene)	F-CCATCCCTATGAGCCA	131 bp	93.2
		R-ACTACTGCCAGCCTGAAGACA		

#### Biochemical analysis of starch

##### Tubers and sample preparation

The harvested tubers from all the mutant lines and wild type Kufri Chipsona-I were washed and chopped into cubes of approximately 1 cm^3^ size and were lyophilized by CHRIST Alpha 1-2 LSC basic lyophilizer to obtain the starch powder. All lyophilized samples were stored in plastic tubes at room temperature until further analysis. As per manufacturer’s instructions, total starch and amylose contents of isolated starch samples were quantified by the Total Starch (AA/AMG) Assay Kit, Amylose/amylopectin kit (Megazyme, Bray, Co, Ireland).

##### Estimation of resistant starch

The resistant starch content of mutated lines along with wild type Kufri Chipsona-I was estimated using a K-RSTAR 06/18 kit from Megazyme (Bray, Ireland), following the AOAC Method 2002.02 (McCleary and Monaghan, 2002). The samples were incubated with pancreatic α-amylase and amyloglucosidase (AMG) (K-RSTAR 06/18; Megazyme, Bray, Ireland) for 16 h at 37 °C. The resistant starch was calculated on a dry weight basis incorporating relative moisture values and following instructions provided in the Megazyme RESISTANT STARCH kit manual, based on the portion of the starch that was not hydrolyzed, and this portion of the starch was taken to be non-resistant (solubilized) starch. The UV-1700 double beam, Spectrophotometer (Shimadzu Corporation, Kyoto, Japan) was used to quantify the resistant starch content at 510 nm in comparison to a blank.

#### Starch structural analysis

##### X-ray diffraction (XRD)

The crystalline structure analysis of starch samples of edited lines and wild type Kufri Chipsona-I was performed using a X-ray diffractometer (XRD-6000, Shimadzu, Brazil), at XRD Laboratory, Panjab University, Chandigarh, India. A target voltage of 30 kV and current of 30 mA was used for the scanned region in the range 5°–30° angles. The scan speed was kept at 1° per minute (2ϴ). XRD-6000 software was used to determine the starch granule’s relative crystallinity (RC). The crystallinity percentage was calculated as per the protocol of López-Rubio et al. (2008).

##### Scanning electron microscopy (SEM)

For visualization of structure, shape and surface morphology of starch granules SEM analysis was done. Starch samples from mutated lines and the wild type Kufri Chipsona-I tubers were taken and frozen at −10 °C. These samples were mounted on circular aluminium stubs then coated with gold and examined under a scanning electron microscope (Model: JSM IT500) using the electron gun having a tungsten filament with an accelerating voltage of 0.3 kV–30 kV at the University Centre for Research and Development (UCRD), Chandigarh University, Punjab, India.

##### ^1^H NMR spectroscopy of starch

Starch from the *SBE* mutated lines (K301- K306) and wild type Kufri Chipsona-I were analysed by Proton nuclear magnetic resonance (^1^H-NMR) to evaluate the degree of branching according to Tizzotti et al. (2024). Total of 10 mg starch samples were dissolved in deuterated dimethyl sulfoxide (DMSO-d6; 600 μL, and deuterated trifluoroacetic acid (TFA-d1; 10 µL) before ^1^H NMR analysis to avoid spectral interference with hydroxyl protons. The ^1^H-NMR spectra were collected from 0 to 7 ppm on an FT-NMR Cryo-magnet Spectrometer 400 MHz (Bruker) at sophisticated analytical instrumentation facility (SAIF) at Panjab University, Chandigarh. Spectra were processed with TopSpin 3.6 at temperature 50 °C using 128 scans and a relaxation delay of 12 s. To measure the degree of branching, the ratio of [H-1 (1→4) + H-l(t) + H-l (1→6)], where I H-1 (1–6) is the integrated signal at 4.77 ppm and I H-1 (1→4) is the integrated signal at 5.12 ppm, corresponding to H^1^ of glucose at the α(1→6) and α(1→4)-linkages, were taken respectively (Gaenssle et al., 2021).

### Statistical analysis

All the experiments were carried out in triplicates and the results were reported as average values with standard deviation (SD). A one-way Analysis of Variance (ANOVA) was performed to analyze all the data using IBM-SPSS version 20. Means were compared using Duncan’s new multiple range test, with significance defined at p ≤ 0.05 (Duncan, 1955).

## Results

### Target identification and sgRNA design

For precise CRISPR-Cas9 mediated editing of *SBE2.1* and *SBE2.2* genes, two sgRNAs targeting exon 1 and exon 2 of *SBE2.1* and two sgRNAs targeting exon 3 of *SBE2.2* were selected. The sgRNAs with highly specificity were chosen based on an out-of-frame score greater than 67 and a GC content ranging between 40% and 60%. These sgRNAs were located adjacent to the protospacer adjacent motif (PAM) sequences and carefully designed to avoid off-targets effects and ensure efficient Cas9-mediated cleavage (Figures 1A,B; Table 1). The designed sgRNAs were subsequently assembled with the *Csy4* gene driven by the CmYLCV promoter and cloned into the pDIRECT_23C vector (Table 2; Figure 1A).

### Optimization of basta selection

**FIGURE 2:**
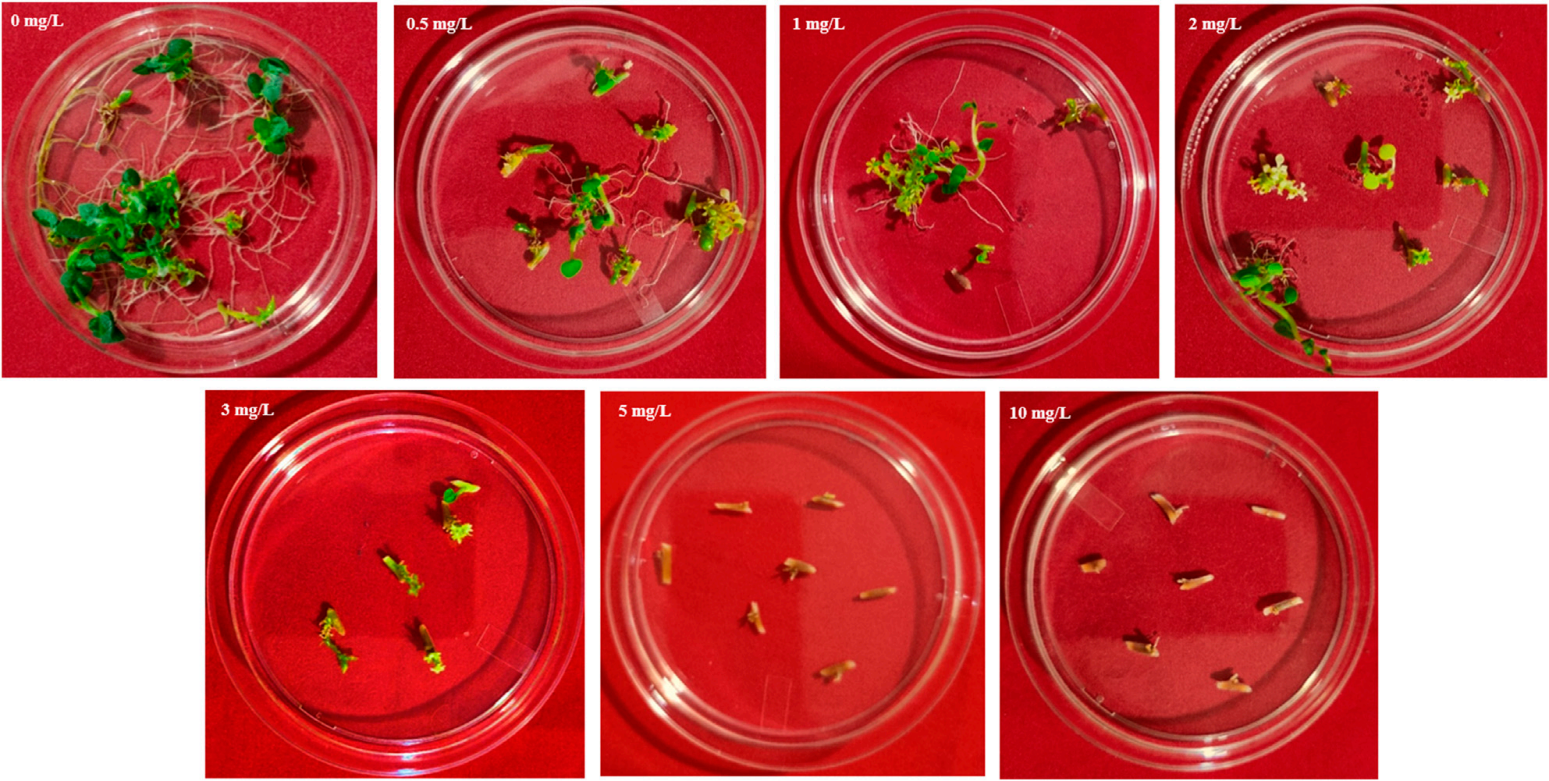
Effect of different concentrations of Basta on Kufri Chipsona-I internodal explants.

The tolerance limit of explants of potato cultivar Kufri Chipsona-I to Basta was examined in the MS medium containing different concentrations of Basta viz. 0, 0.5 mg/L, 1.0 mg/L, 2.0 mg/L, 3 mg/L, 5.0 mg/L and 10 mg/L respectively. In the absence of Basta, callus induction and shoot organogenesis were observed in 95% of the explants. Furthermore, the survival rate of explants drastically reduced with an increase in Basta concentration, and all the explants on the medium containing 5 mg/L Basta died (Figure 2). Therefore, with increasing levels of Basta selection, 5 mg/L was found to be the optimum threshold concentration for callus induction and shoot organogenesis. Thus, 5 mg/L of Basta was used in subsequent genetic transformation experiments for the selection of putative transformants.

### *Agrobacterium-*mediated plant transformation & regeneration of putative transformed lines

**FIGURE 3:**
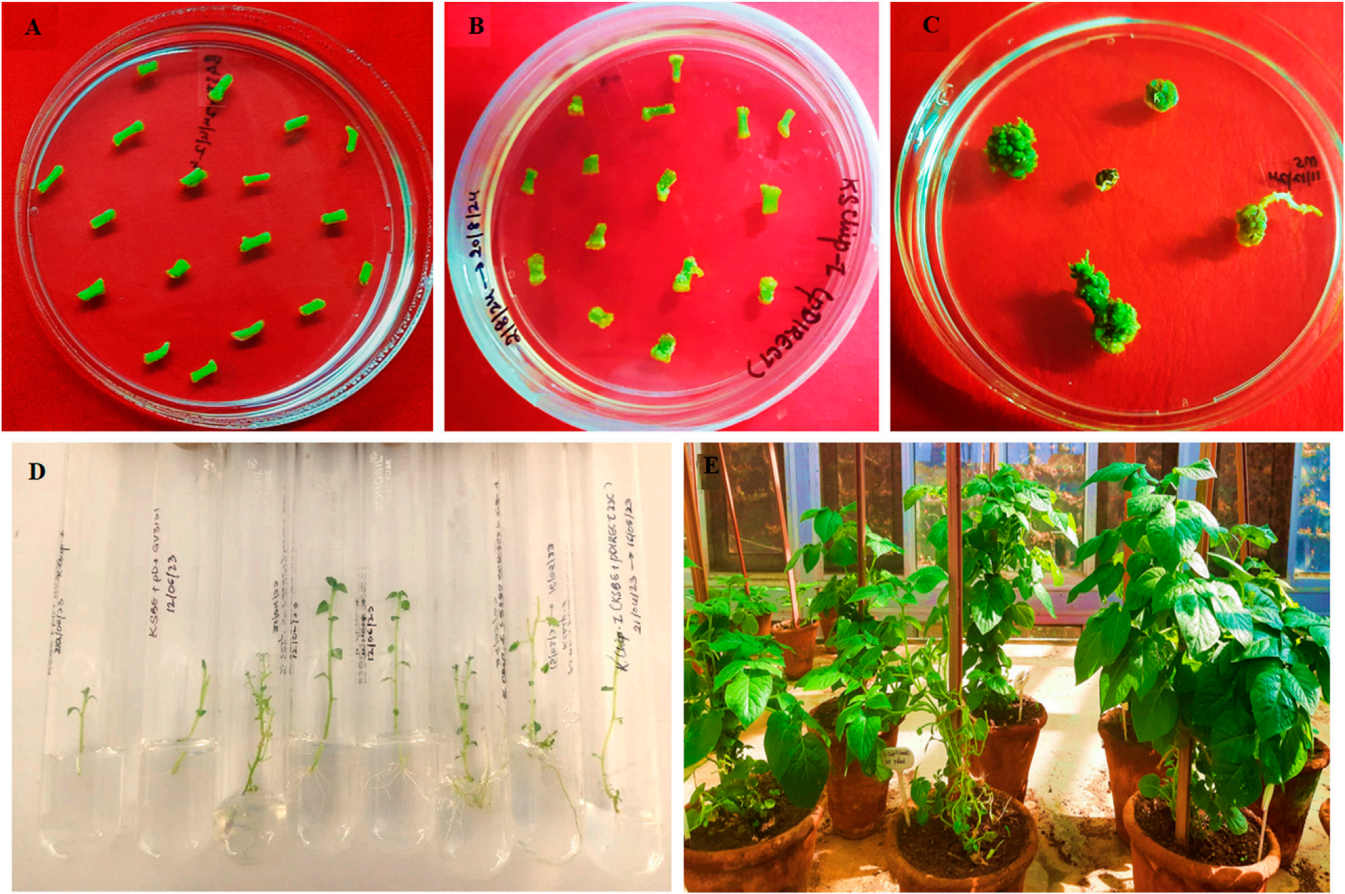
Genetic transformation of Kufri Chipsona-I with *A. tumefaciens* harbouring *SBE*-pDIRECT23C construct; **(A)** internodal explants cultured on MS medium, **(B)** callus induction and proliferation, **(C)** shoot regeneration, **(D)** shoot elongation and rooting, **(E)** hardened transformed plants.

The internodal segments obtained from wild-type Kufri Chipsona-I were transformed with *A. tumefaciens* harbouring *SBE*-pDIRECT23C construct for targeting the *SBE2.1* and *SBE2.2* genes. The 2-day co-cultivation with 100 μM acetosyringone was found to be most effective for transformation and 88.6% internodal segments showed callus induction (Figure 3). A total of 55.5% regeneration frequency was observed, and the regenerated shoots multiplied and elongated on the same medium. Further, *in vitro* regenerated transformed plantlets were hardened in the glasshouse for tuber development, achieving 100% survival rate.

### Molecular confirmation of edited lines

**FIGURE 4:**
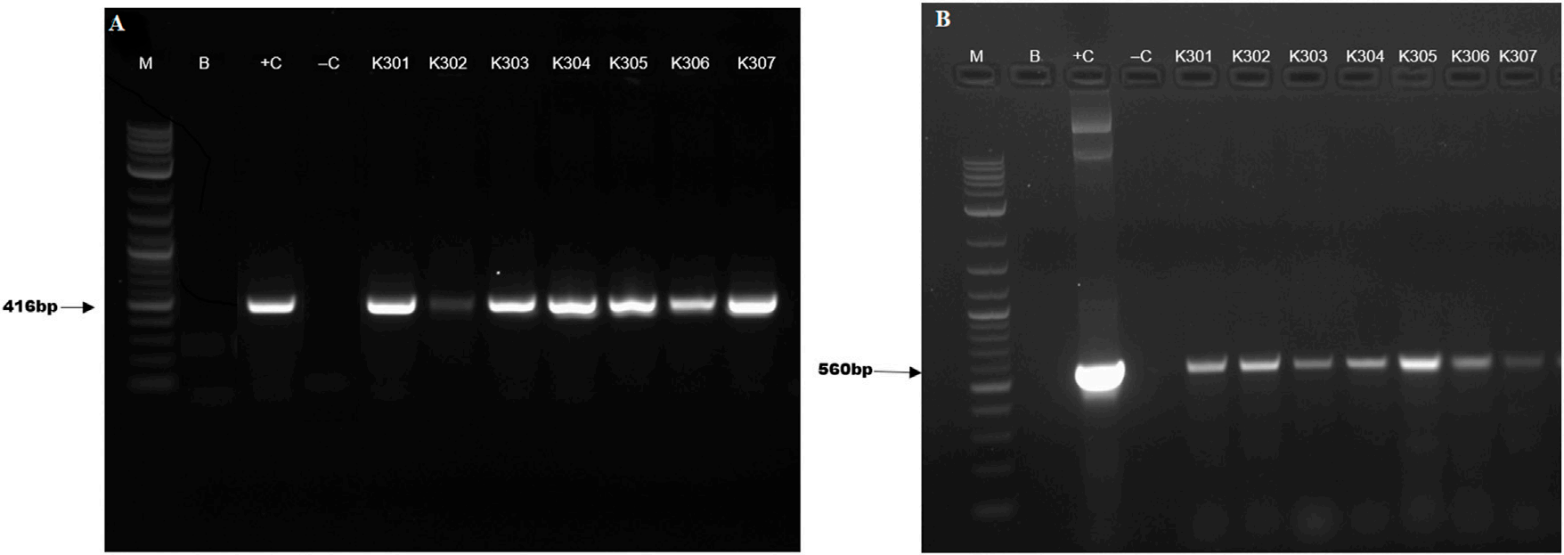
PCR screening of transformed plants carrying (A) *bar* and (B) *Cas9* genes, where, lane M-1 Kb plus ladder, B-Blank, +C-positive control, -C- negative control and K301, K302, K303, K304, K305, K306 represent putative edited events.

Successful T-DNA integration in the putative edited/transformed potato lines had been confirmed by PCR amplification of *Cas9* and *bar* genes. PCR amplification yielded bands of size 560 and 416 bp respectively for *Cas9* and *bar* genes in all the transformed potato lines (Figure 4).

### T7EI assay

**FIGURE 5:**
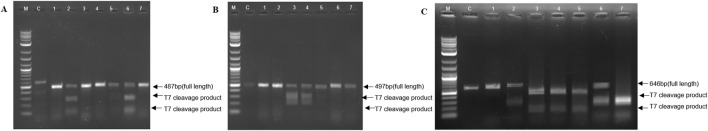
T7 endonuclease I (T7E1) assay for detection of CRISPR-Cas9-induced mutations at the **(A)** SBE2.1 (target 1) **(B)** SBE2.1 (target 2), and **(C)** SBE2.2 gene targets (target 1 & 2), where lane M-1 Kb plus ladder, C-control, 1- Untreated DNA, and lanes 2-7 represent K301, K302, K303, K304, K305, K306 putative edited events.

The efficiency of the *SBE*-pDIRECT23C construct in inducing mutations at desired target sites in the *SBE2.1* and *SBE2.2* genes was estimated by using the T7E1 assay. Only six transformed potato lines showed digestion of the PCR products of target sites with T7E1 and were defined as mutant lines (Figures 5A–C). The digested DNA fragments from K301, K304, K305 lines at *SBE2.1* (target 1) and K302, K303, and K304 lines at *SBE2.1* (target 2) showed heteroduplex formation. Similarly, K301, K302, K303, K304, K305, and K306 lines showed endonuclease-driven fragment cleavage at both target amplicons of *SBE2.2* (targets 1 and 2) (Figures 5A–C). The mutation frequency of 50% was obtained in the undigested PCR product as compared to the T7E1-digested product.

### Sanger sequencing of target gene fragments

**FIGURE 6:**
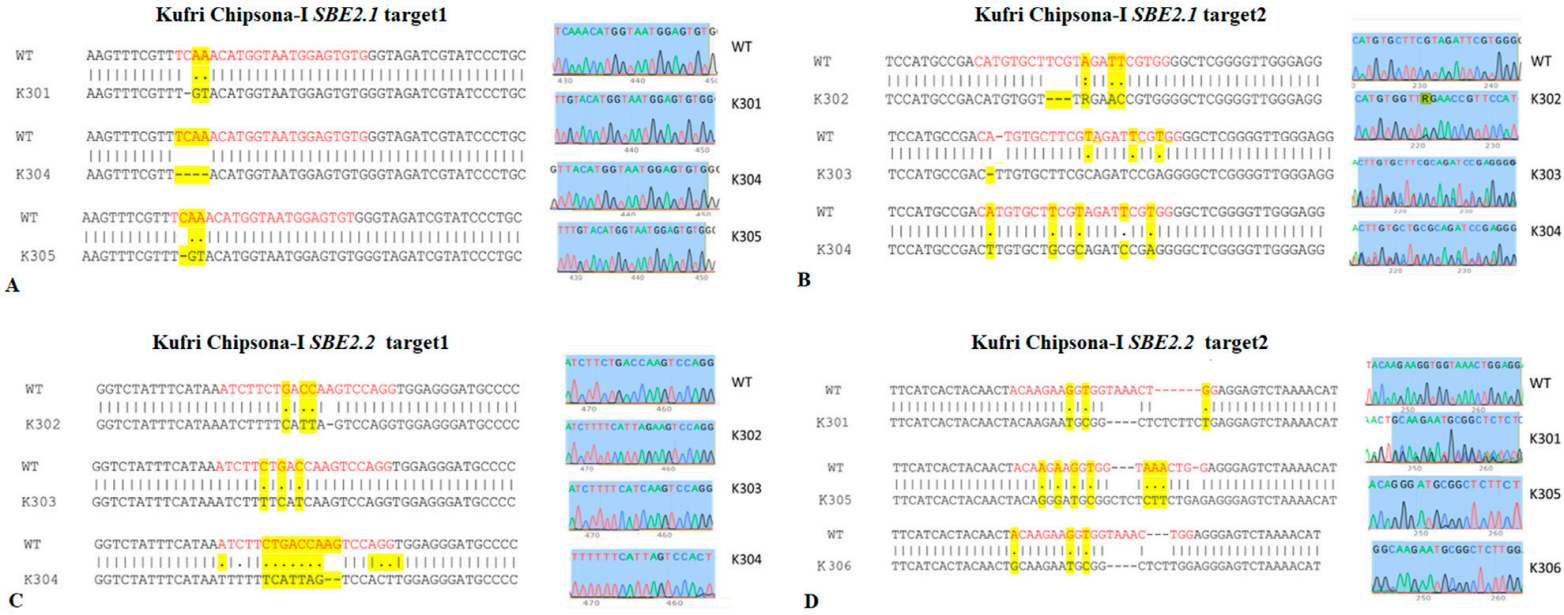
Sequence alignments showing CRISPR-Cas9 induced mutations in SBE2.1 & SBE2.2 genes (target 1 & target 2) **(A)** SBE2.1 (target 1) **(B)** SBE2.1 (target 2) **(C)** SBE2.2 (target 1) **(D)** SBE2.2 (target 2). The sgRNA target sequences are shown in red colour. The indels and substitutions are highlighted in yellow, where deletions are represented by dashes ‘‐’ and substitutions by ‘.’

**FIGURE 7:**
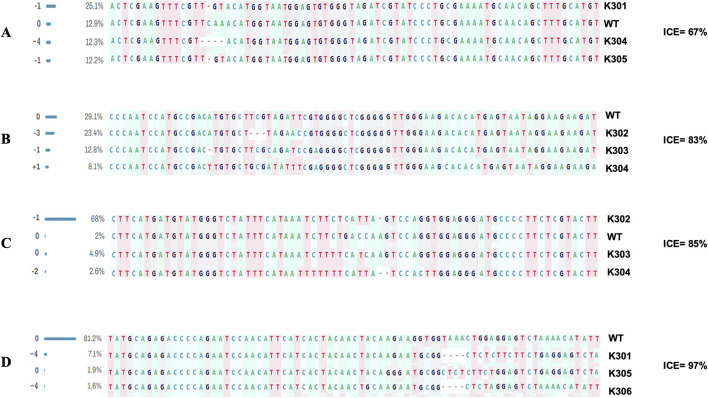
Indel mutations analysis at target sites in *SBE2.1* & *SBE2.2* genes using Synthego ICE software **(A)** *SBE2.1* (target 1) **(B)** *SBE2.1* (target 2) **(C)** *SBE2.2* (target 1) **(D)** *SBE2.2* (target 2).

Sequence analysis of both target sites of *SBE2.1* and *SBE2.2* genes, from the wild type Kufri Chipsona-I and six mutant lines (K301-K306) showed that mutants have visible indels as well as base pair substitution in the target regions (Figure 6) where substitution and deletions are more frequent types of mutation. Sequence analysis of *SBE2.1* gene (target 1) showed that three lines K301, K304, and K305 have base pair substitution as well as indels. In contrast, the Sanger sequencing results of *SBE2.1* gene target 2 showed both indels & substitutions of bases in edited lines K302, K303, and K304. The observed base pair substitutions and indels in *SBE2.2* target sites across different lines (K301, K302, K303, K304, K305 and K306) demonstrated the functional activity and editing precision of the CRISPR-Cas9 system. The type of mutations and editing efficiencies analysed using Synthego ICE Analysis tool revealed indel frequencies of ICE 67% for *SBE2.1* (target 1), ICE 83% for *SBE2.1* (target 2), ICE 85% for *SBE2.2* (target 1), and ICE 97% for *SBE2.2* (target 2). The edited potato lines exhibited both indel and substitution mutations, which were present at comparable frequencies across the target gene loci (Figure 7). Overall, the K304 line exhibited the highest frequency of indels and substitutions in three targets across both selected genes, thus showed the highest level of genome editing efficiency among the edited lines.

**FIGURE 8:**
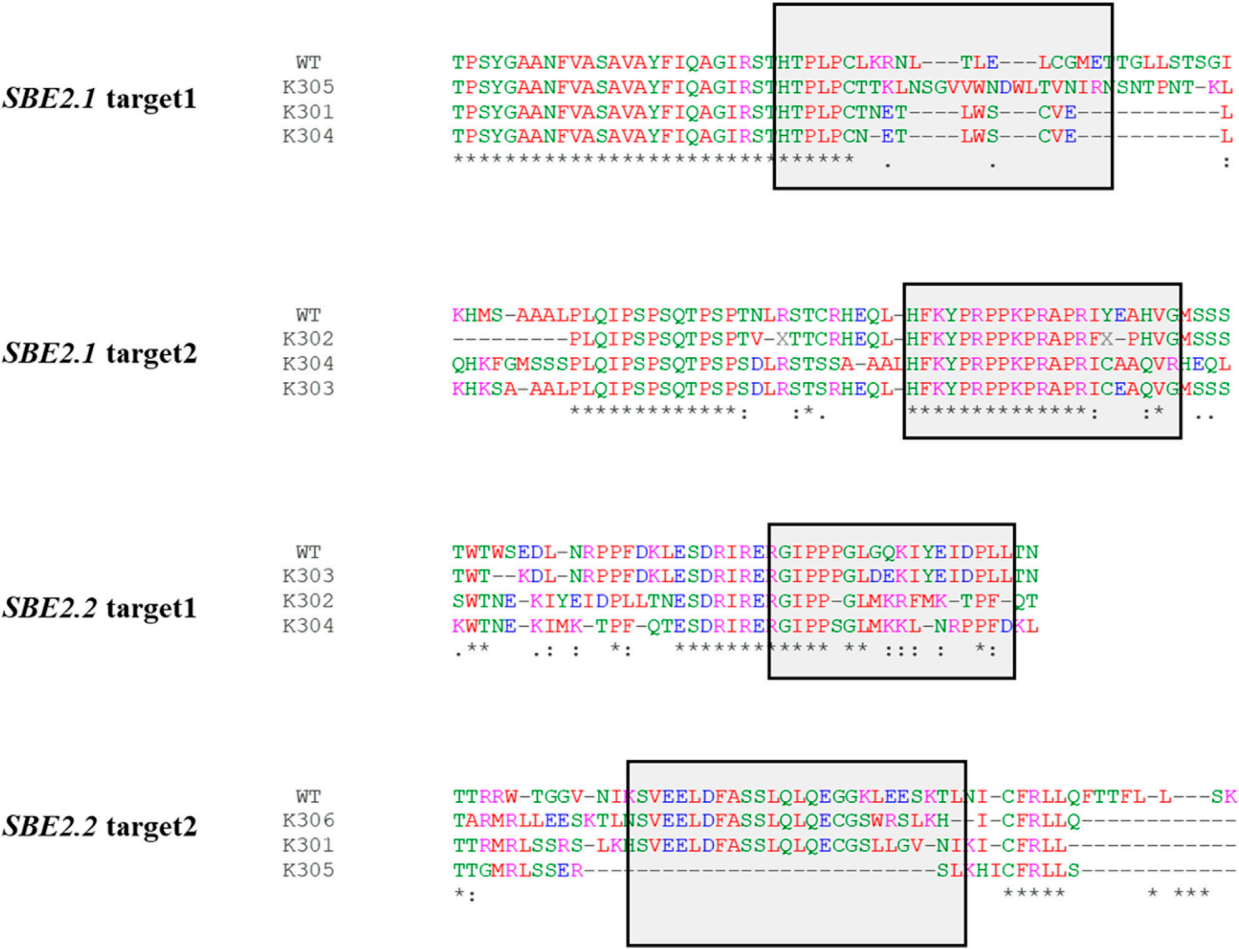
Translated sequence of the regions of the *SBE2.1* & *SBE2.2* genes (target 1 & target 2) of the wild-type and the edited events showing changes in the amino acid at the target cleavage site and the get cleavage site and the introduction of PSCs.

*In silico* protein translation of *SBE2.1* and *SBE2.2* gene sequences of the wild-type Kufri Chipsona-I and mutated lines was done using the Expasy-Translate tool. The translated sequence alignment for target 1 of *SBE2.1* gene revealed the frame shift mutation in K305 and premature stop codons (PSCs) in K301 and K304 edited lines. Target 2 of the *SBE2.1* gene showed a frame shift mutation in K303 and K304, and PSCs in K302 edited lines. Similarly, for the *SBE2.2* gene, K303, K304 showed a frame shift mutation in target 1, while PSCs were observed in K302 (target 1) and K301, K305, K306 (target 2) mutated lines (Figure 8).

### qRT-PCR analysis

**FIGURE 9:**
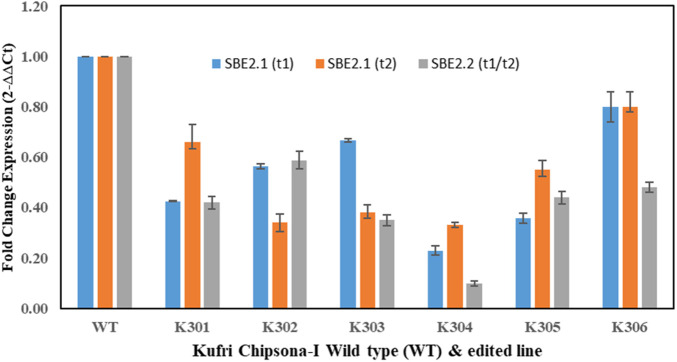
qRT-PCR gene expression analysis of *SBE2.1* & *SBE2.2* (target 1 & 2) in mutant (K301-K306) and wild type Kufri Chipsona-I. The asterisks (*) and (**) marks indicate statistically significant difference (p ≤ 0.01) and (p ≤ 0.05).

The gene expression analysis of the *SBE2.1* and *SBE2.2* genes in all six mutated lines and wild type Kufri Chipsona-I was done by performing qRT-PCR. The decreased mRNA expression of *SBE2.1* (target 1) gene was observed in all six mutated lines and wherein, the K301, K304 and K305 mutant lines showed the significantly low mRNA expression levels of 0.42, 0.23, 0.36 folds, compared to the wild type Kufri Chipsona-I. Similarly low levels of *SBE2.1* (target 2) gene expression was recorded in all six mutant potato lines and out of these the lowest gene expression folds of 0.34, 0.38, 0.33 was recorded in K302, K303 and K304 lines. The decreased fold expression of the *SBE2.2* (target 1 and 2) gene, *i.e*., 0.42, 0.58, 0.35, 0.10, 0.44 and 0.48 was found in K301, K302, K303, K304, K305 and K306 edited lines, respectively, in comparison to the unmutated wild type Kufri Chipsona-I (Figure 9). qRT-PCR analysis revealed a significant reduction in the expression of both *SBE2.1* and *SBE2.2* genes across all six mutated potato lines compared to the wild-type Kufri Chipsona-I. The extent of downregulation varied among specific mutants, with some showing the lowest expression levels, confirming that Cas9-induced mutagenesis altered gene sequences and consequently suppressed gene expression.

### Starch content and its structural analysis in mutant lines of potato

The quantification of total starch, resistant starch, and amylose in mutant lines (K301, K302, K303, K304, K305, and K306) and the wild type Kufri Chipsona-1 revealed significant biochemical variations. These changes are likely to have resulted from underlying genetic modifications affecting starch biosynthetic and degradative pathways. The total starch content was observed, in a range of 82.93%–83.02% as compared to the wild type Kufri Chipsona-1 (84.62%). Resistant starch levels varied significantly among the edited lines, with the highest in K304 (8.69%) and the lowest in the wild type Kufri Chipsona-1 (4.89%) (Table 4). Amylose content displayed the widest variation among all parameters, ranging from 27.11% in the wild type Kufri Chipsona-1–95.91% in K304 (Table 4).

**TABLE 4:** Estimation of total starch, resistant starch, and amylose content in edited potato lines.

S. No.	Edited potato lines	Resistant starch (RS) (g/100 g)	Amylose (%)
1	K301	6.57 ± 0.41^c^	55.53 ± 0.37^c^
2	K302	8.48 ± 0.39^days^	59.44 ± 0.49^e^
3	K303	8.44 ± 0.46^days^	82.20 ± 0.41^f^
4	K304	8.69 ± 0.29^days^	95.91 ± 0.48^g^
5	K305	7.04 ± 0.15^c^	57.94 ± 0.32^days^
6	K306	5.74 ± 0.28^b^	35.17 ± 0.58^b^
7	Wild type Kufri Chipsona-I	4.89 ± 0.22^a^	27.11 ± 0.48^a^

### Physiochemical analysis of starch granules using SEM, XRD and ^1^H NMR spectroscopy

**FIGURE 10:**
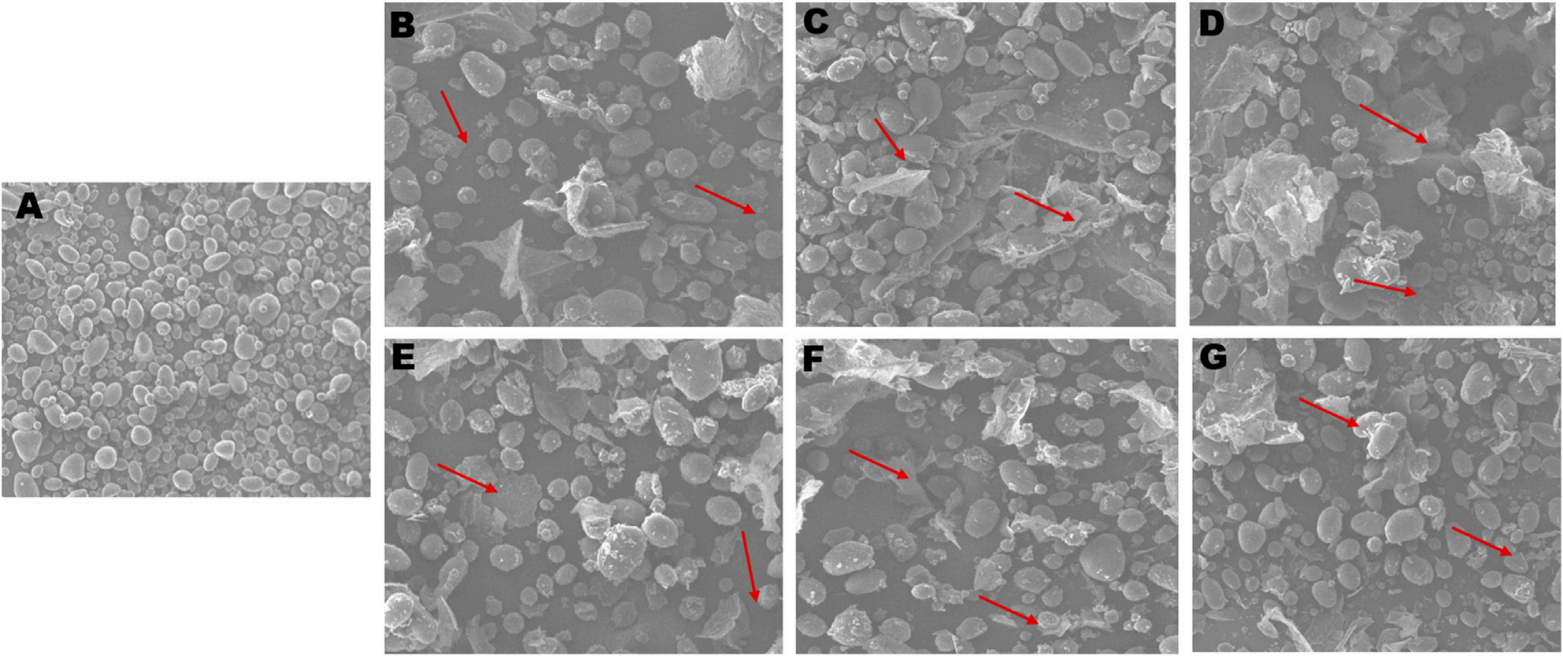
SEM analysis of starch granules **(A)** wild type Kufri Chipsona-I; **(B-G)** mutant lines (K301-K306). Red arrows illustrate the nodulation and distorted granules.

The surface morphology of starch granules in wild type Kufri Chipsona-I tuber was observed to have smooth granules without cracks or granulations and unaltered growth rings. While the starch obtained from *SBE2.1* and *SBE2.2* mutated lines has irregularities in the in the starch granules morphology, with a rough surface and amorphous regions. All the six CRISPR-Cas9 edited lines showed the alteration in appearance of starch granules, which hints at the insufficient amount of amylopectin, which is required for a smooth surface of starch granules (Figure 10). However, the tuber starch content is found to be statistically similar to that of wild type Kufri Chipsona-I tuber starch.

**FIGURE 11:**
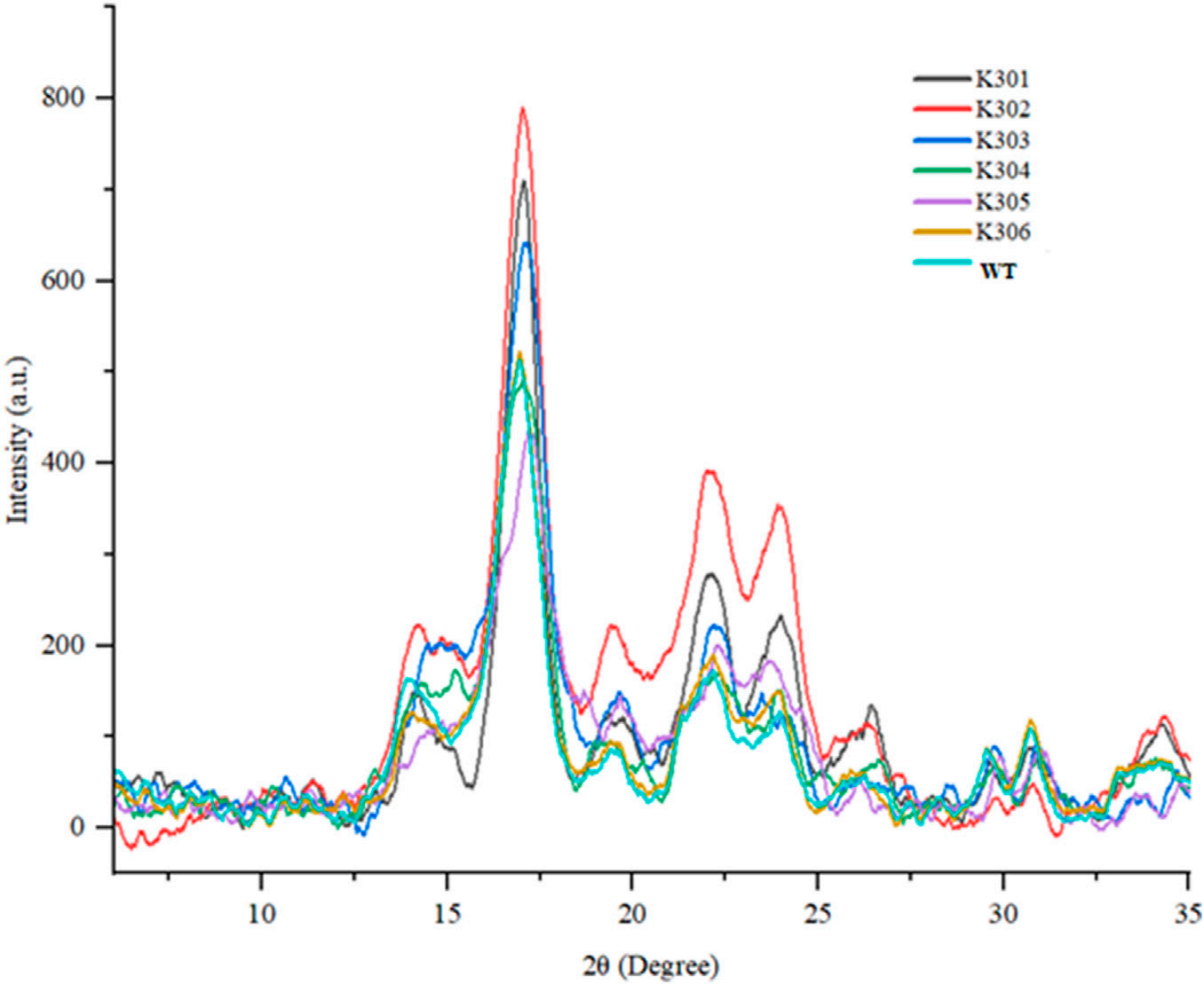
X-ray diffraction patterns of mutant (K301-K306) and Wild-type Kufri Chipsona-I potato lines.

The XRD results revealed the characteristic B-type crystallinity, with strong peaks at ∼17° and a doublet at 23°–25° and significant differences in starch crystallinity among the *SBE2.1* and *SBE2.2* gene-edited lines in comparison to wild-type Kufri Chipsona-I (Figure 11). Higher diffraction intensities were observed in K301, K302, and K303 edited lines, which indicated more ordered granular structures, likely due to double-helical packing of amylopectin, which is proportional to the overall crystallinity. In contrast, K304, K305, and K306 edited lines exhibited lower intensities, suggesting disrupted crystallinity. The crystallinity index (%) calculated for the edited lines has varied to a significant extent showing lowest 24.27% in K302% and 28.87% in wild type Kufri Chipsona-I (Table 5).

**TABLE 5:** Crystallinity degree (CI %) of starches from edited potato lines (K301- K306 and wild type Kufri Chipsona-I).

Sr No.	Edited potato lines	CI %	Crystal pattern
1	K301	28.29 ± 0.52^c^	B-type
2	K302	24.27 ± 1.10^a^	B-type
3	K303	28.45 ± 0.32^c^	B-type
4	K304	28.6 ± 0.01^c^	B-type
5	K305	26.16 ± 0.21^b^	B-type
6	K306	25.61 ± 0.07^b^	B-type
7	Wild type Kufri Chipsona-I	28.87 ± 0.06^c^	B-type

**FIGURE 12:**
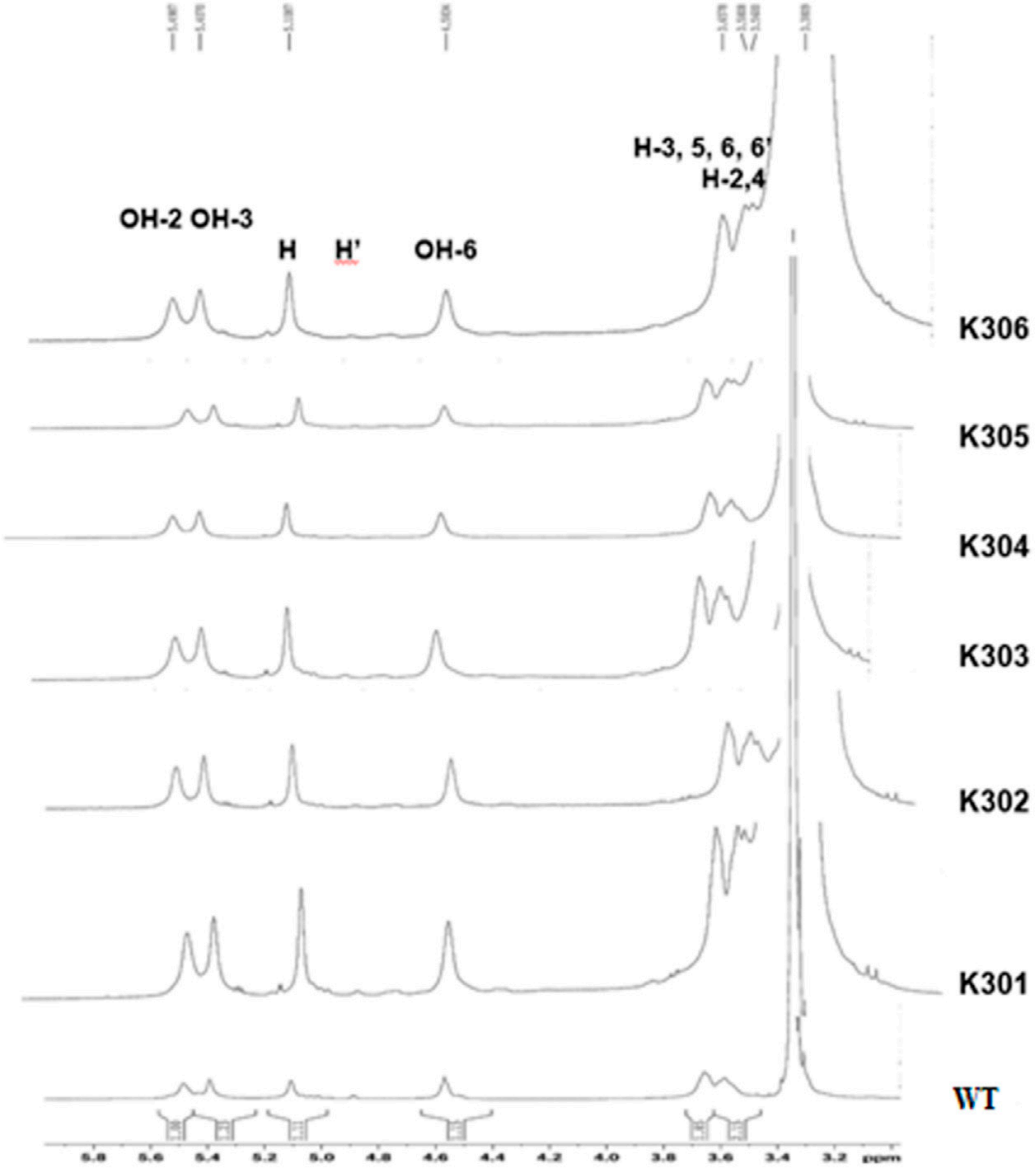
^1^H NMR spectra of solubilized starch showing α-1,4 and α-1,6 linkages in mutant lines (K301-K306) and wild type Kufri Chipsona-I.

In the present study, the signature peak position of wild type Kufri Chipsona-I starch dissolved in deuterated DMSO (DMSO-d_6_) solvent showed the strong peak at 2.50 ppm, while the peaks corresponding to anhydroglucose were identified at 5.60–5.54 ppm (O–H protons at positions 2 and 3), 3.60 ppm (protons at positions 3/5/6/6′/6″), and 3.30 ppm (protons at positions 2/4/). In ^1^H NMR, the anomeric protons involved in α-1,4 and α-1,6 linkages have been used to estimate the average degree of branching of d-glucans. The peaks at 5.20–5.10 ppm represent the proton on the linear α-1,4 linkages, and at 4.9 ppm represent the proton at the branch point (α-1,6 linkages), respectively. ^1^H NMR analysis indicated that the branch points of α-1,6-glycosidic bonds in amylopectin component of starch from the mutated potato lines have slightly been reduced relative to the wild-type starch (Figure 12). The degree of branching calculated from the peaks observed in *SBE2.1* and *SBE2.2* edited potato lines showed lower values in a range of 1.15%–3.66%, in comparison to the wild type Kufri Chipsona-I which was recorded to be 5.46% (Table 6).

**TABLE 6:** Estimation of degree of branching (%) in edited potato lines.

S.No.	Edited potato lines	H-1 (1 → 4)	H- 1 (t)	H-1 (1 → 6)	CL_n_	Degree of branching (%)
1	K301	1.26	2.82 + 4.17	0.3	8.55	3.45 ± 0.10 ^days^
2	K302	1.43	1.89 + 2.55	0.15	6.02	2.50 ± 0.12^c^
3	K303	1.26	2.52 + 3.30	0.1	7.18	1.46 ± 0.01^b^
4	K304	1.01	2.48 + 3.42	0.08	6.99	1.15 ± 0.06^a^
5	K305	1.45	2.88 + 3.41	0.3	8.04	3.66 ± 0.15^e^
6	K306	1.09	2.08 + 2.54	0.2	5.91	3.43 ± 0.06^days^
7	Wild type Kufri Chipsona-I	1.11	1.85 + 2.15	0.3	5.41	5.46 ± 0.12^f^

## Discussion

In India, the increasing prevalence of obesity, diabetes, and associated health complications has increased the demand for potatoes enriched with dietary fiber. The resistant starch high in the prebiotic dietary fiber content provides a diabetic-friendly alternative that has a low glycemic index, boosts overall gut health and is also useful in the starch-based food and potato chips industry for improving texture and nutritional value. Therefore, in this study, *SBE2.1* and *SBE2.2* genes have been targeted for the first time in an Indian potato cultivar Kufri Chipsona-I, using CRISPR-Cas9-mediated targeted mutagenesis to generate high-amylose and resistant-starch lines. The two sgRNAs for each isoform were designed for precise and accurate mutagenesis of *SBE2.1* and *SBE2.2* genes. Targeting two sites for site-directed mutagenesis leads to higher frequency of deletions compared to the fewer indels observed when a single site is targeted (Cermak et al., 2017).

In the present study 5 mg/L Basta concentration was found to be optimum for selection of positive transformants. The effective dosage of Basta herbicide is species dependent and has been reported to range between 0.2 mg/L to 50 mg/L for inhibiting the growth and regeneration of different non-transformed plants (Bakhsh et al., 2020; Lakhani and Tiwari, 2025). The callus induction was observed 2 weeks after *Agrobacterium*-mediated genetic transformation, which is consistent with previous reports, where callus formation typically begins within 10–14 days under optimal conditions (Awasthi et al., 2022). Overall, 55.5% callus have shown shoot regeneration. These findings are in line with earlier reports where 45% shoots were regenerated from the transformed callus of Kufri Chipsona-I (Siddappa et al., 2023).

PCR amplification of the *bar* and *Cas9* gene was performed to confirm the presence of sgRNA expression cassette. Results showed that 56% of positive transformants exhibited successful and stable integration in the host plant Kufri Chipsona-I. Various studies have demonstrated this PCR-based strategy as a rapid and efficient method for identifying transformed plants harboring both *Cas9* and *bar* marker genes (Nadakuduti et al., 2018). Further, these positive lines were analysed for mutation detection using T7EI assay. The mutation frequency of 50% was observed in the present study, which is in line with the mutation frequency of 52%–72% reported in *SBE1* and *SBE2* mutant potato lines using the CRISPR-Cas9 method (Zhao et al., 2021). Similarly, T7EI based mutation analysis studies have reported 71.2% and 76.9% mutation frequencies in *Arabidopsis* and tomato, respectively (Feng et al., 2014; Pan et al., 2016). These results suggest that T7E1 sensitivity is highly influenced by the complexity of indels present in the mutated cells (Sentmanat et al., 2018).

Furthermore, the Sanger sequence analysis of all the obtained mutant lines has shown visible indels as well as base pair substitutions in the target region. Similarly, Zhao et al. (2021) have also reported in-frame indels in *SBE1* and *SBE2* mutated potato lines. The editing efficiency and mutation type among the lines may be attributed to sequence-specific factors, including GC content of sgRNA, which is well-known to influence its stability, Cas9 binding, and cleavage efficacy. All the mutated lines have exhibited mutations at distinct target sites within the same gene, underscoring the multiplexing potential of CRISPR-Cas9 in targeting multiple loci. The mutation analysis using ICE software has shown high indel frequencies across the target sites, reflecting the efficient genome-editing activity of the designed sgRNAs. Among all the six mutated Kufri Chipsona-I lines, the K304 line, has shown the mutation in both target 1 and target 2 of *SBE2.1* and target 1 of *SBE2.2,* thus showing maximum number of overall indels in the genome at target sites. In contrast, all other edited lines exhibited mutations in at least two target sites. The target 1 and target 2 of *SBE2.2* exhibited the highest editing efficiency (85% and 97%), indicating that this locus may possess favourable sequences and structural features that enhance Cas9 accessibility and cleavage efficiency. The ICE algorithm showed that all four sgRNA target sites were mutated in obtained six mutant lines, suggesting efficient sgRNA selection and Cas9-mediated cleavage. The ICE results are consistent with other findings in potato crop showing that, even within the same gene or genomic region, editing efficiencies can vary significantly among target sites, likely due to differences in local DNA sequences and chromatin structure (Andersson et al., 2018; Chincinska et al., 2022).

In the present study, a qRT-PCR analysis revealed that the expression levels of *SBE2.1* and *SBE2.2* genes were significantly lower in all edited lines compared to the unedited wild-type Kufri Chipsona-I. The lower expression levels of *SBE2.1* and *SBE2.2* genes, suggested that gene editing has influenced starch metabolism in these edited lines, possibly leading to higher amylose content. In a similar study, editing of *SBE2.1* and *SBE2.2* genes in potato has shown altered amylopectin structure and a higher amylose-to-amylopectin ratio (Zhao et al., 2021). Li et al. (2023) have also reviewed that the downregulation of *SBE* genes would lead to modified starch production for increased dietary fiber.

The total starch content was found to be similar in all the *SBE2*.*1* and *SBE2*.2 mutated lines as well as wild type Kufri Chipsona-I. These results suggested that the proportion of amylose and amylopectin may have changed, while the total starch composed of glucose units remains almost the same (Smith et al., 2017). The difference in total starch content reflects either downregulation of these biosynthetic genes or increased activity of starch-degrading enzymes such as amylases or glucan water dikinases (*GWD*) (Martens et al., 2018). Elevated resistant starch in mutant line K304 (8.69%) is likely to arise from structural changes in starch granules, particularly due to the increased amylose content. In a similar study, high resistant starch was reported in *SBE2* mutant Cassava plant with 56% high amylose content (Luo et al., 2022), and also 53.48% high amylose was reported in Cas9-mediated *SBEIIb* edited gene in maize (Ma et al., 2024). Zhao et al. (2021) reported a high (89%) amylose producing *SBE1* and *SBE2* mutant potato lines. Whereas, a 40% increase in amylose content was reported in *SBE*-mutated potato lines (Jayarathna et al., 2024). The altered amylopectin branching and greater amylose-lipid complex formation, have been reported to reduce its accessibility to digestive enzymes and thereby suggesting it as a key determinant of resistant starch formation (Reddy et al., 2021).

The substantial increase in resistant starch among these mutants, especially in K304, has proved it as a strong candidate for health-oriented applications, such as low-glycemic index foods and functional starch-based products developing functional foods targeting metabolic health. The SEM analysis of all the *SBE2*.1 and *SBE2*.2 mutated lines showed deformities, granulation of large size granules, and rough outer surface of starch granules. The weak packaging of amylopectin chains and poor integrity of starch molecules would have led to these deformities in starch granules. Kaul et al. (2023) have also demonstrated that the chemically modified starches may lead to structural changes and irregularities in pre-gelatinized starches.

The XRD study of all the samples exhibited characteristic B-type crystallinity, with strong peaks at ∼17° and a doublet at 23°–25°, consistent with tuber starches, which is in line with earlier studies on potato starch (Lian et al., 2017). The CI% values of the potato starches of edited lines ranged from 24.81% to 28.90%, compared to the wild type Kufri Chipsona-I (28.90%). These results have suggested that edited lines retained a highly ordered double-helical structure of amylopectin, characteristic of B-type crystallinity normally found in tuber starches. Similarly, Zhao et al. (2021) have also reported a decline in the diffraction intensity for most of the B-type starch crystal peaks in *SBE1* and *SBE2* mutated lines. High crystallinity typically correlates with greater molecular packing and resistance to enzymatic digestion, which supports their potential as sources of resistant starch (Martens et al., 2018). In contrast, potato mutant lines such as K302 (24.81%), K305 (26.10%), and K306 (25.69%) displayed relatively lower CI% values, indicating partial disruption of the crystalline regions. However, the CI% values in the mutant lines did not differ much when compared to wild type Kufri Chipsona-I, possibly due to the freeze-drying of samples before XRD analysis as described in the study by Harris and Warren. (2024).

The ^1^H NMR study revealed that the edited lines have shown a reduced degree of branching that resulted in short amylopectin chains. These observations are in corroboration with the similar Cas9-mediated mutations generated in the potato *SBE2* gene (Tuncel et al., 2019). Mutants with strong reductions in the degree of branching in starch significantly influence its physicochemical properties, affecting its functionality in various applications (Wang et al., 2022). More branching indicates a more branched amylopectin structure, leading to increased solubility and altered gelatinization behaviour. Conversely, a lower degree of branching suggests a more linear structure, typically associated with higher amylose content, resulting in different functional properties such as increased tendency for retrogradation (Li et al., 2023). These results substantiate the fact that starches with a higher degree of branching tend to have more surface area, which makes them more prone to enzymatic digestion, unlike amylose, which shows a low degree of branching. Thus, the CRISPR-Cas9-mediated editing of *SBE2.1* and *SBE2.2* genes in potato has significantly increased amylose and resistant starch content in the potato cultivar Kufri Chipsona-I, which is the utmost need of potato-based industries.

## Conclusion

The targeted mutagenesis of *SBE2.1* and *SBE2.2* genes in Kufri Chipsona-I using the CRISPR-Cas9 system has successfully generated high-amylose potato lines with significantly increased resistant starch content. Sanger sequencing revealed indel mutations at the target sites which was found to be associated with elevated resistant starch and amylose contents in the potato cultivar Kufri Chipsona-I. This study has suggested a functional link between the targeted *SBE2.1* and *SBE2.2* mutations and resistant starch production. Structural analysis confirmed the presence of B-type crystallinity, reduced branching, and disrupted starch granule morphology, indicative of altered starch composition. These genome-edited lines developed in our study using the CRISPR-Cas9 system represent the first Indian potato variants developed through precise gene editing for enhanced nutritional quality and offer valuable genetic material for breeding programs focused on health-promoting traits.

## Data Availability

The original contributions presented in the study are included in the article/supplementary material, further inquiries can be directed to the corresponding authors.
